# Evaluation of accuracy of various impression techniques and impression materials in recording multiple implants placed unilaterally in a partially edentulous mandible- An *in vitro* study

**DOI:** 10.4317/jced.54726

**Published:** 2018-04-01

**Authors:** G. Parameshwari, B. Chittaranjan, N. Sudhir, C.-K. Anulekha-Avinash, M. Taruna, M. Ramureddy

**Affiliations:** 1 MDS, Assistant professor, Department of Prosthodontics, Kamineni Institute of Dental Sciences, Nalgonda, Telangana, India; 2 MDS, Principal, Department of Prosthodontics, Mallareddy Institute of Dental Sciences, Hyderabad, Telangana, India; 3 MDS, Professor, Department of Prosthodontics, Kamineni Institute of Dental Sciences, Nalgonda, Telangana, India; 4 MDS, Associate professor, Department of Prosthodontics, Kamineni Institute of Dental Sciences, Nalgonda, Telangana, India; 5 MDS, Professor and Head, Department of Prosthodontics, Kamineni Institute of Dental Sciences, Nalgonda, Telangana, India; 6 MDS, Professor, Department of Prosthodontics, Kamineni Institute of Dental Sciences, Nalgonda, Telangana, India

## Abstract

**Background:**

Various factors like implant angulation, type of impression material and tray type affects the implant impression accuracy. To date limited *in-vitro* studies were carried out on the implant impression accuracy of unilateral partially edentulous arches. The aim of this research was to evaluate the effects of 0o, 15o and 25o implant angulations on impression accuracy in simulated master casts of unilateral partially edentulous situation using different impression materials and tray selections.

**Material and Methods:**

30 replicas (N = 30) of a resin matrix (control) containing four implant analogues placed unilaterally from the midline till the region of second molar at an angulation of 00, 00, 150 and 250 to the vertical axis of the ridge respectively were obtained by using three impression techniques (stock metal tray, closed custom tray, and open nonsplinted custom tray) and two different impression materials (Polyvinyl-siloxane and polyether). Specific dimensions of the resultant casts were measured using coordinated measuring microscope. Mean linear changes were calculated and statistically analyzed using analysis of variance (ANOVA) and Tukey’s post-hoc procedures (*p*< 0.05).

**Results:**

The casts obtained from all three impression techniques had significant differences in dimensions (*p*<0.05) as compared to that of master model irrespective of impression materials. Comparing the techniques with regard to the parallel implants, no statistical significant difference (*p*<0.05) was observed with custom tray techniques (closed/open). Whereas while comparing parallel versus non parallel, open tray technique showed superior accuracy compared to closed tray technique as the angulation increased more than 15 degrees.

**Conclusions:**

The influence of material and technique appeared to be significant for highly non axial implant angulations (*p*< 0.05), and increased angulation tended to decrease impression accuracy. The open tray technique was more accurate with highly nonaxially oriented implants for the small sample size investigated.

** Key words:**Implant impressions, partially edentulous arch, angulated implants.

## Introduction

Osseointegrated implants are used for the rehabilitation of complete and partially edentulous patients. Accuracy of the impression procedure is an utmost important factor for the success of the implant prosthesis. A variety of factors may affect the accuracy of implant impressions such as different impression techniques (1,2), impression materials, tray type (3), the number of implants, angulation of implants or abutments (4) and prosthetic connection features (5).

Literature reveals limited information about the impression accuracy of partially edentulous arch with multiple non parallel implants compared to completely edentulous arch.

The purpose of this in vitro study was to compare the accuracy outcomes of the nonsplinted open and closed-tray implant impression techniques using two different elastomeric impression materials with straight and angulated implants placed unilaterally in a partially edentulous mandibular model.

## Material and Methods

Master model fabrication

**Figure 1:**
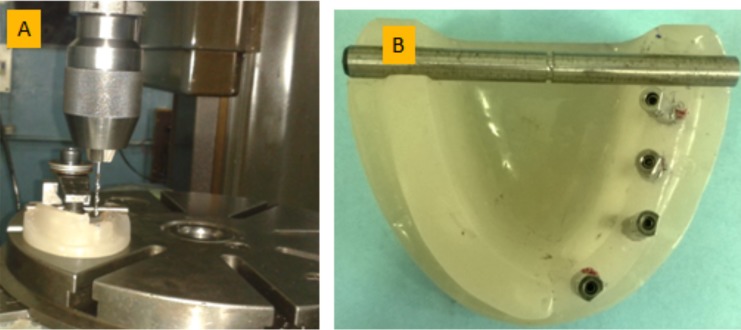
Horizontal milling machine is used for drilling the master model. 1B) Resin master model is showing implant placement.

To simulate a clinical scenario of implant supported prosthesis a heat-cure clear acrylic resin model of a partially edentulous mandible (DPI heat cure denture base polymer) was fabricated. Stainless steel metallic insert (ᴪ6×70mm dowel) was placed in the posterior region of the resin model, which was used as the standard reference for measuring the position of the implant replica. Using a horizontal milling machine four implant sites were machined 6mm apart equidistantly, two in anterior region and other two in posterior region respectively (Fig. 1A). The two anterior implant sites were machined parallel to each other and perpendicular to the horizontal plane. Two more implant analogues were machined at an angulation of 15 and 25 degrees to the long axis tilting lingually at the posterior sites. Four internal hex implant analogs (ADIN dental implants, Israel) 3.75 mm in diameter and 10 mm in length were secured in position using auto polymerizing acrylic resin (DPI cold cure denture base polymer) (Fig. 1B).

The types of impression trays used were (i) metal stock trays (ii) closed custom trays (iii) open window custom trays.

Two types of impression materials (polyvinyl Siloxane and polyether) were used for making impressions. These materials were grouped as Group I (Polyvinyl Siloxane); Group II (Polyether). Each group further divided into three subgroups based on impression techniques ( Table 1). Five impressions were made for each subgroup and finally a total of thirty impressions (N=30) were made.

**Table 1:**
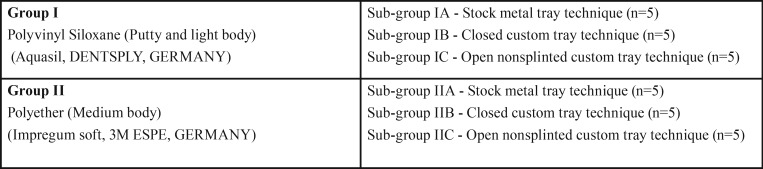
Grouping of the samples.

**Figure 2:**
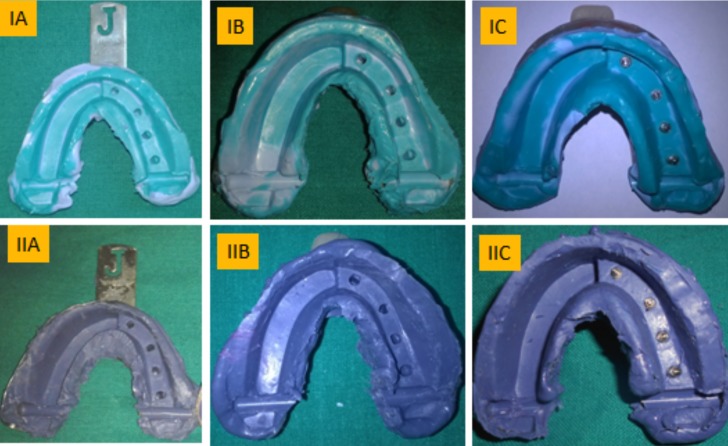
A) Polyvinyl siloxane impressions made using (IA) Stock tray, (IB) Closed custom tray and (IC) Open non splinted custom tray. B) Polyether impressions made using (IIA) Stock tray, (IIB) Closed custom tray and (IIC) Open nonsplinted custom tray.

In Group I – Impressions were made using polyvinyl Siloxane impression material (Aquasil, Dentsply) using a putty-wash single step technique (Fig. 2A). Both in sub group IA and sub group IB closed tray transfer copings were connected to the implant analogues of the master model. Tray adhesive (Universal tray adhesive, Zermack, Italy) is applied on inner surface of the tray and allowed to dry for 15 min before making an impression. In subgroup IC open nonsplinted custom trays were used. During the impression making the excess material on the top of the impression coping was removed to expose the upper portion of impression coping. In Group-II, Impressions were made using medium bodied consistency polyether impression material (3M ESPE), while the impression procedure and the trays used in the sub-groups were similar to sub-groups of Group I (Fig. 2B).

The closed tray impressions were separated from the resin master model leaving behind the impression copings. Subsequently the transfer copings were detached from the master model and attached to the laboratory analogues, and then the coping-analogue assemblies were positioned before pouring the impression to a definitive cast.

Whereas in open tray nonsplinted impression technique after impression material was set the screws were unscrewed and the impression copings were picked along with the impression. Impression copings were secured with laboratory analogs before pouring the impressions.

The polyvinyl siloxane impressions were poured after 2 hours according to the manufacturer’s instructions. The polyether impressions were poured after 1 hour to simulate clinical situation. 100 gms of Type IV dental stone was mixed with 22 ml of distilled water using vacuum mixer (Wehmer) and casts were derived by following manufactures instructions. The casts were allowed to set for 1 hour before retrieving from the impression. The casts were subjected to measurement after 24 hours in order to simulate clinical situation.

**Figure 3:**
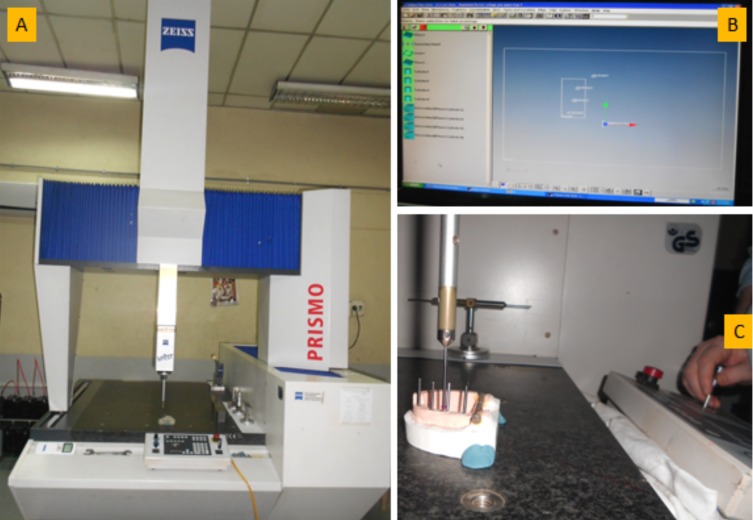
A) Coordinated Measuring Microscope (CMM) with 1mm wide straight probe, B) Data processor, 3C) Long guide screws were tightened into the implants to reveal central axes of the implants.

Measurements:

Deviations of the models were analyzed using coordinated measuring microscope (PRISMO, ZIESS) (Fig. 3A). It has a 1mm wide straight probed sensor which is capable of measuring in X-, Y-, Z- axes with an accuracy of ± 5µm. While measuring, the microscope was connected to a data processor (Fig. 3B). The measurements of the master model were made to provide the reference to be compared with the experimental casts. Long guide screws of open tray transfers were tightened into the implant analogues of the master model in order to reveal the central axes of each implant analogue (Fig. 3C).

**Figure 4:**
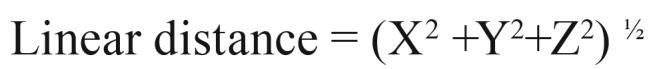
Formula.

All the measurements were made in reference to posterior reference plane. Then the position of implant analogue 1 was measured from the midpoint of the reference plane which was measured by using straight probe of microscope to get the distance between metallic insert and analogue 1. After the implant analogue 1 was located in X, Y and Z axes, it was used as the reference to locate the position of other three analogues. The distance between analogue 1 and 2, 1 and 3, and 1 and 4 were given by the data processor. In X, Y and Z co-ordinates linear distance can be calculated by using this formula (6), (Fig. 4).

**Figure 5:**
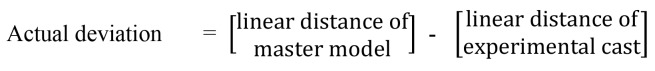
Formula.

The linear distance of the master model and experimental models were calculated by using above mentioned formula. Then the linear distance of experimental models were subtracted from the corresponding linear distance of master model, to get the actual deviation in millimeters, (Fig. 5).

Statistical analyses:

Data was statistically analyzed by one way analysis of variance (ANOVA) followed by multiple comparison Post Hoc Tukey-HSD. Same level of significance (*p* < 0.05) was used throughout the study.

## Results

The accuracy of three different impression techniques with two different impression materials of partially edentulous arches with multiple angulated implants was compared. A total of 30 casts were made from group I (polyvinyl siloxane) and group II (polyether). Each group is further divided into three subgroups containing five casts each. In the group I (polyvinyl siloxane) experimental casts obtained using stock tray impression technique exhibited more deviations (0.1077, 0.1672 and 0.1971) ( Table 2) and less deviations (0.0624, 0.0960 and 0.1005) were observed with open unsplinted custom tray impression technique as the implant angulation increases from the midline to posterior region compared to master cast. Similar results were found in group II (polyether) using stock tray impression technique (0.1126, 0.1706 and 0.1993) and open custom tray impression technique (0.0631, 0.0965 and 0.1040) respectively. The post hoc Tukey’s test ( Table 3, Table 4) indicated that irrespective of impression material, open nonsplinted tray impression technique exhibited improved accuracy compared to closed custom technique with parallel implants (A2) but not statistically significant (*p* <0.05) and showed statistically significant results with non parallel implants (A3 and A4).

**Table 2:**
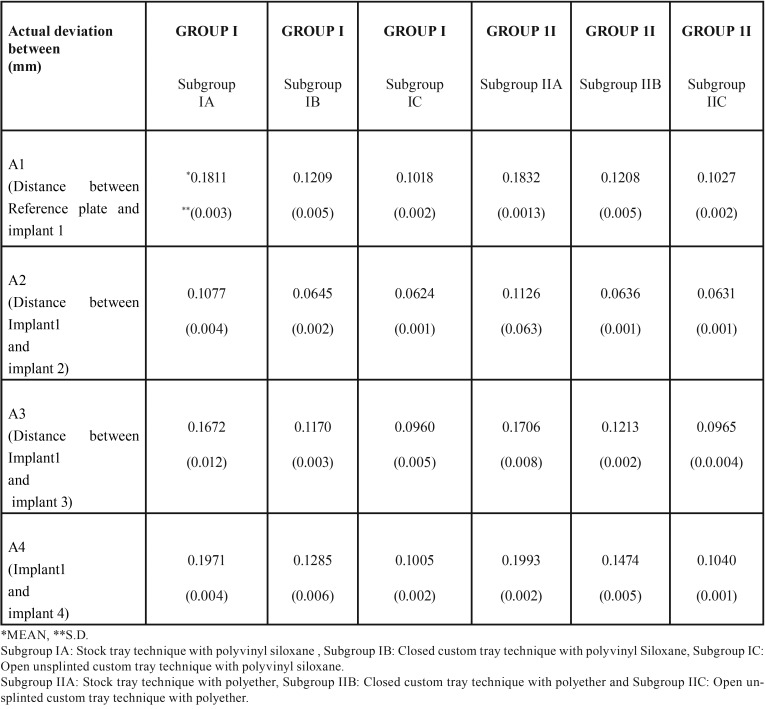
Summary statistics according to three impression techniques in Group I (Polyvinyl siloxane) and Group II (Polyether) with respect to distances A1, A2, A3 and A4 respectively.

**Table 3:**
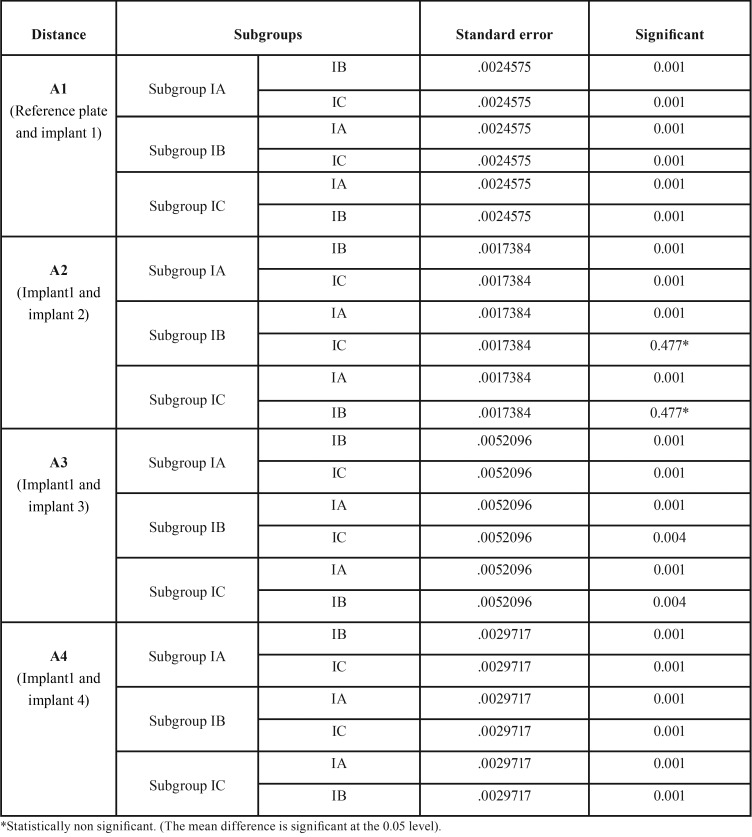
Pair wise comparison of three impression techniques with respect to distances A1, A2, A3 and A4. (Mm) by Tukeys HSD Post Hoc procedures in Group I (Polyvinyl Siloxane).

**Table 4:**
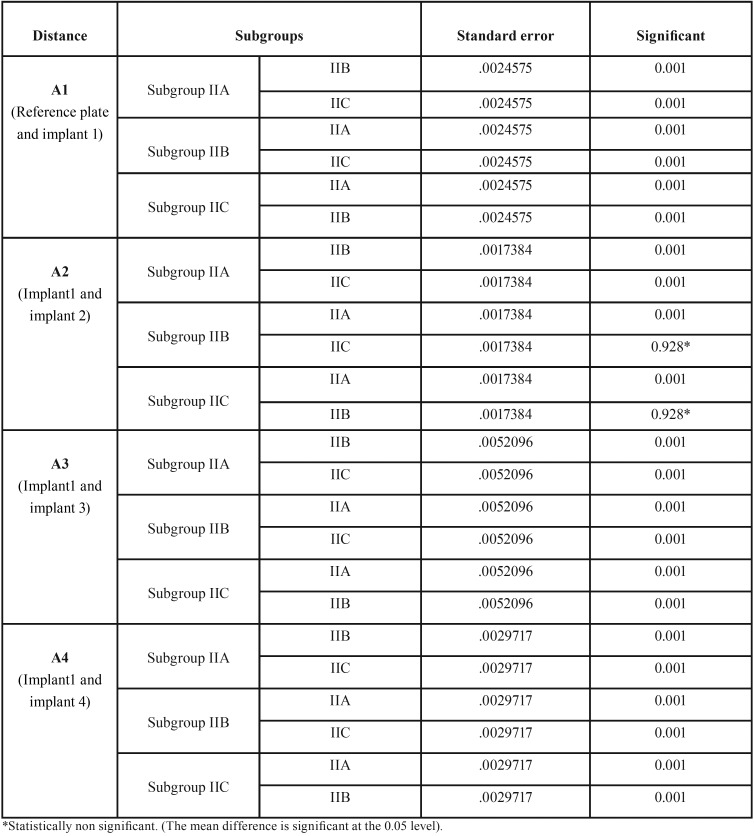
Pair wise comparison of three impression techniques with respect to distances A2, A3 and A4 (Mm) by Tukey HSD Post Hoc procedures in Group II (Polyether).

## Discussion

In clinical scenarios, it is sometimes difficult to obtain an exact parallel placement of implant due to the limitations of the anatomical structures. The angulations of these implants can range from 5 degrees to 40 degrees (7) such a scenario faces with difficulties in obtaining accurate impressions. Controversies still exist as to which impression technique can prove to be best in such cases. Hence this study was conducted to compare and evaluate impression accuracy with various implant angulations (00, 150 and 250) using stock trays, closed and open non splinted custom trays using polyvinyl siloxane and polyether impression materials.

The present study revealed that, greatest mean linear dimensional distortion resulted from impressions made with the stock tray technique compared to custom tray technique using polyvinylsiloxane and polyether ( Table 3, Table 4). These values obtained were statistically significant (*p* < 0.01) and are similar to results of studies obtained by Del’acqua MA *et al.* (8) and Rupali Patil *et al.* (9).

One clinical study (10) reported no difference in the clinical accuracy between open unsplinted and closed custom tray impression techniques for partially edentulous patients with two implants and up to 10 degrees angulation. Carr (11) reported that angulation up to 15 degrees had no effect on impression accuracy, while Jang *et al.* (12) reported that angulation greater than 20 degrees negatively affected the accuracy. When implant angulation was 30 degrees, Howell *et al.* (13) reported that the open unsplinted custom tray technique was more accurate than closed custom tray. Similar to previous studies open unsplinted custom tray impressions showed better accuracy in comparison with closed custom tray impressions in relation to linear dimensional change between non parallel implants (A3 and A4) ( Table 3) and this change showed statistical significance (*p* < 0.01). In contrast to the results obtained in present study there are studies which have conducted by Cehreli MC and Akça K reported that closed custom tray was more accurate compared to open unsplinted tray technique for partially edentulous patients (14).

In the present study polyvinyl Siloxane impressions showed less distortion compared to polyether for both closed and open nonsplinted technique using custom trays. Polyvinyl Siloxane impressions obtained using closed custom tray technique showed superior accuracy and statistical significant difference (*p*< 0.01) when compared with polyether impressions of a partially edentulous arch with non-parallel implants (A3 and A4) ( Table 2). Similar to present study Sorrentino *et al.* (15) observed accurate impressions with polyvinyl siloxane impression material compared to polyether impression material.

The use of the polyether in a partially edentulous arch could lead to an increased difficulty for a removal of the impression due to its rigidity and presence of undercuts. The addition silicones, because of the lower and more favorable modulus of elasticity, could be considered as a viable alternative allowing for the easy removal of the impression and reducing the permanent deformations caused by the stress between the impression material and the copings, particularly when nonparallel implants are present especially in closed tray technique (16).

A systematic review (17) investigated the accuracy of all published implant impression techniques and examined the clinical factors affecting impression accuracy. The study concluded that there was no statistically significant difference in the accuracy of pick-up non splinted and transfer techniques when there were three or fewer implants, but the pick-up technique produced superior accuracy for multiple implants with implant angulation more than 20 degrees. Similar to previous studies present study also showed that pick-up non splinted technique produced less distortion compared to transfer technique when more than three implants were used.

## Conclusions

Within the limitations of the present study, irrespective of impression material open non splinted custom tray technique showed statistically significant difference compared to closed custom tray technique for the multiunit partially edentulous situation, where the inter implant distance increases and implant angulation increases from 15 to 25 degrees. For closed tray technique, as the inter implant distance increases along with the increase in the implant angulation it was noted that polyvinyl siloxane recorded less distortion than polyether.
